# Differential Distribution of the *wlaN* and *cgtB* Genes, Associated with Guillain-Barré Syndrome, in *Campylobacter jejuni* Isolates from Humans, Broiler Chickens, and Wild Birds

**DOI:** 10.3390/microorganisms8030325

**Published:** 2020-02-26

**Authors:** Pedro Guirado, Sonia Paytubi, Elisenda Miró, Yaidelis Iglesias-Torrens, Ferran Navarro, Marta Cerdà-Cuéllar, Camille Stephan-Otto Attolini, Carlos Balsalobre, Cristina Madrid

**Affiliations:** 1 Departament de Genètica, Microbiologia i Estadística, Facultat de Biología, Universitat de Barcelona. Avda. Diagonal 643, 08028 Barcelona, Spain; pedro.guirado.frias@gmail.com (P.G.); s.paytubi@gmail.com (S.P.); cbalsalobre@ub.edu (C.B.); 2 Hospital de la Santa Creu i Sant Pau and Institut d’Investigació Biomèdica Sant Pau (IIB Sant Pau), Sant Quintí 89, 08041 Barcelona, Spain; EMiro@santpau.cat (E.M.); yaide87@yahoo.com (Y.I.-T.); 3 Departament de Genètica i Microbiologia. Universitat Autònoma de Barcelona, 08193 Cerdanyola del Vallès (Barcelona), Spain; 4 IRTA, Centre de Recerca en Sanitat Animal (CReSA-IRTA-UAB). Campus de la Universitat Autònoma de Barcelona, Bellaterra, 08193 Barcelona, Spain; marta.cerda@irta.cat; 5 Institute for Research in Biomedicine (IRB Barcelona), The Barcelona Institute of Science and Technology, Baldiri Reixac 10, 08028 Barcelona, Spain; camille.stephan@irbbarcelona.org

**Keywords:** *Campylobacter*, lipooligosaccharide, *wlaN*, *cgtB*, Guillain-Barré syndrome

## Abstract

*Campylobacter jejuni* causes campylobacteriosis, a bacterial gastroenteritis with high incidence worldwide. Moreover, *C. jejuni* infection can trigger the polyneuropathic disorder denominated Guillain-Barré syndrome (GBS). The *C. jejuni* strains that can elicit GBS carry either *wlaN* or *cgtB,* coding both genes for a β-1,3-galactosyltransferase enzyme that is required for the production of sialylated lipooligosaccharide (LOS^SIAL^). We described a differential prevalence of the genes *wlaN* and *cgtB* in *C. jejuni* isolates from three different ecological niches: humans, broiler chickens, and wild birds. The distribution of both genes, which is similar between broiler chicken and human isolates and distinct when compared to the wild bird isolates, suggests a host-dependent distribution. Moreover, the prevalence of the *wlaN* and *cgtB* genes seems to be restricted to some clonal complexes. Gene sequencing identified the presence of new variants of the G- homopolymeric tract within the *wlaN* gene. Furthermore, we detected two variants of a G rich region within the *cgtB* gene, suggesting that, similarly to *wlaN*, the G-tract in the *cgtB* gene mediates the phase variation control of *cgtB* expression. Caco-2 cell invasion assays indicate that there is no evident correlation between the production of LOS^SIAL^ and the ability to invade eukaryotic cells.

## 1. Introduction

*Campylobacter jejuni* causes the most common bacterial food-borne disease in Europe, a gastroenteritis named campylobacteriosis, which can range from mild, watery to bloody diarrhea [1,2]. Campylobacteriosis is mostly transmitted to humans by the consumption and improper handling of poultry meat [3]. Wild birds play a relevant role in the *C. jejuni* transmission route by being carriers that can infect both poultry and humans [4,5]. Moreover, campylobacteriosis is the most frequent infection preceding Guillain-Barré syndrome (GBS), an acute polyneuropathic disorder [6]. The strains of *C. jejuni* that can potentially elicit GBS are those that produce a sialylated LOS (LOS^SIAL^), due to the well-documented molecular mimicry between the LOS^SIAL^ and the saccharide component of the human GM1 ganglioside which is present in peripheral nerves [7]. Antibodies generated against *Campylobacter* LOS^SIAL^ structures might cross-react with GM1 found in nerve tissue [1]. Consistently, during the acute-phase of *C. jejuni*-associated GBS infection, the sera contain high titers of antibodies against LOS^SIAL^ that cross-react with GM1 gangliosides [8].

The operon responsible for the biosynthesis of LOS is highly diverse among *C. jejuni* isolates, with up to 19 different locus classes, named from A to S [9,10]. Only classes A, B, and C carry genes coding for a β-1,3-galactosyltransferase catalyzing the addition of a galactose molecule, which is required for the production of a GM1-like LOS^SIAL^ structure [11]. Two different genes have been identified to encode for this enzymatic activity: *cgtB* (in class A and B) and *wlaN* (in class C) [10,12]. The amino acid identity between CgtB and WlaN is about 58% [12].

Genomic analyses revealed the presence of hypervariable sequences in *C. jejuni* genes coding for proteins involved in the biosynthesis of surface structures [13]. Most of these hypervariable sequences consist of short homopolymeric nucleotide tracts. The *wlaN* gene contains a G-tract located within the coding sequence, rendering the expression of the *wlaN* gene under phase variation control, presumably by a slipped-strand mechanism [12]. Three different G-tract variants have been described, carrying either six, eight, or nine residues [12,14]. The presence of an 8G-tract results in a full-length product (ON-phase), whereas either a 6G- or a 9G-tract results in truncated proteins (OFF-phase).

In this work, we describe the prevalence of the genes responsible for LOS^SIAL^ production, *cgtB* and *wlaN*, in *C. jejuni* isolates from different ecological origin: humans, broiler chickens, and wild birds. The presence and diversity of homopolymeric G-tract regions within both LOS^SIAL^-related genes are studied. The correlation between the production of LOS^SIAL^ and the ability to invade eukaryotic cells is evaluated.

## 2. Materials and Methods

### 2.1. Bacterial Strains and Culture Conditions

The *C. jejuni* strain collection, previously described [15], is composed of 150 isolates obtained from faeces of three different sources: human patients suffering from symptomatic gastroenteritis (50 isolates), broiler chickens (50 isolates), and wild birds (50 isolates). Human isolates were obtained from the Santa Creu i Sant Pau Hospital (Barcelona) strain collection. Broiler chicken isolates belong to a *Campylobacter* strain collection at IRTA-CReSA, collected at different slaughterhouses located in Barcelona, Lleida, and Tarragona (Catalonia). The wild bird samples, collected in Catalonia and Alboran Island, were obtained from six different species (*Spatula clypeata*, *Ciconia ciconia*, *Corvus corax*, *Columba livia*, *Larus michahellis,* and *Larus audouinii*). The strain 81-176 (ATCC BAA-2151) was used as the reference strain [13]. Complete genomes of *Campylobacter jejuni* strains were recovered from an NCBI database.

Isolates were cultured onto Columbia blood agar (CBA) plates (Scharlau) and incubated at 42 °C for 48 h in microaerophilic conditions (CampyGen, Oxoid), unless otherwise stated.

### 2.2. PCR Amplification

Genomic DNA was extracted from cultures grown onto CBA plates using the InstaGene Matrix Kit (Bio-Rad Laboratoires). PCR reactions (PCR Master Mix x2, Thermo Scientific) were performed using 35 ng of DNA as a template and the presence of *wlaN* and *cgtB* genes were tested using the specific primers indicated in Table 1. Primers used to amplify the housekeeping gene *gltA* were included in the PCR mixtures as an internal control of the PCR reaction. Amplified fragments of *wlaN* and *cgtB* were purified and sequenced with the same primers that were used in the PCR reaction.

### 2.3. Analysis of LOS

Cell biomass from one CBA plate was resuspended in 1 ml of PBS and the OD_600_ of the cell suspension determined. After centrifugation, the cells were resuspended in Laemmli buffer, adjusting the final volume accordingly to the OD_600_ of the cell suspension (OD_600_ × 0.5) (whole cell extracts). LOS was isolated as described previously [18]. Briefly, samples were incubated at 100 °C for 10 min and centrifuged at 10,000× *g* for 10 min. A 50 µl aliquot of the supernatant was treated with proteinase K (20 mg/ml; 2 h at 65 °C). LOS samples were fractionated by 15% SDS-PAGE and visualized by a carbohydrate specific silver staining method [16]. LOS^SIAL^ was detected by Western blot using a peroxidase-conjugated labelled cholera toxin B (HRP-CT), as previously described [12]. Coomasie staining of whole cell extracts was used as the loading control.

### 2.4. Invasion Assay

Adherence and invasion assays were performed using human colonic carcinoma (Caco-2) cells, as previously described [19]. Caco-2 cells were seeded in 24-well plates at 2 × 10^4^ cells per well and incubated for 8 days at 37 °C. Bacteria, grown on CBA plates under microaerophilic conditions for 24 h at 37 °C, were resuspended in PBS plus 1% inactivated-FBS (PBS-F) and the bacterial concentration was adjusted at approximately 2 × 10^8^ cfu/ml (OD_600_ of 0.04). Confluent monolayers of Caco-2 cells were washed once with PBS and infected with 0.5 ml of the bacterial suspension. To allow bacterial adherence and internalization, monolayers and bacteria were incubated for 3 h at 37 °C and 5% CO_2_ in a humified atmosphere. For total cell-associated bacteria (intracellular and adhered) quantification, the unbound bacteria were removed from cell monolayers by washing with PBS, the cells were lysed with 0.5 ml Triton X-100 (1%) for 10 min, and the total cell-associated bacteria was determined by serial dilutions on CBA plates. For intracellular bacteria quantification, infected monolayers were washed with PBS and incubated in 0.5 ml of fresh PBS-F with 150 ml/ml gentamycin (Sigma) to kill extracellular bacteria. After 2 h at 37 °C, cells were washed and lysed following the same procedure as for the total cell-associated bacteria. The amount of both intracellular and total cell-associated bacteria (intracellular and adhered) was determined in triplicate assays. The invasion index was calculated as the percentage of intracellular bacteria relative to the total cell-associated bacteria.

## 3. Results

### 3.1. Differential Prevalence of wlaN and cgtB Genes in C. jejuni Isolates from Human Patients, Broiler Chickens, and Wild Birds

**Figure 1:**
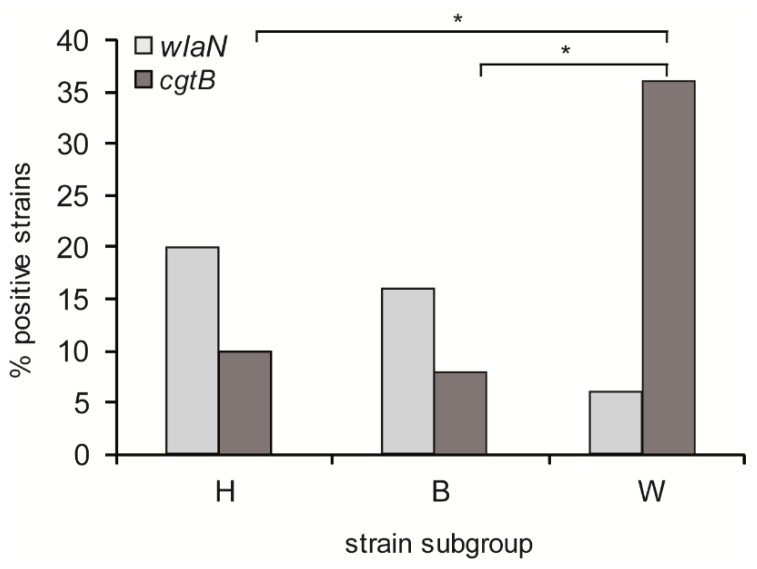
Prevalence of *wlaN* and *cgtB* genes in *C. jejuni* strains from different origins. H, human patients; B, broiler chickens; W, wild birds. Statistical analyses were performed using Pearson’s chi-squared test (R Studio software). *p* < 0.005 was considered statistically significant (indicated by an asterisk).

The presence of the *wlaN* and *cgtB* genes in a collection of 150 *C. jejuni* strains from human patients (50), broiler chickens (50), and wild birds (50) was determined by PCR. A similar percentage of strains were positive for LOS^SIAL^ related genes (*wlaN*^+^ and *cgtB*^+^) among human (28%) and broiler chicken (22%) strains. The percentage increased to 40% among wild bird strains. Interestingly, more striking differences exist in the prevalence of each specific gene depending on the origin of the strains (Figure 1). The *wlaN* gene was more frequently detected among human and broiler chicken strains (20% and 16%, respectively) than the *cgtB* gene (8% and 6%, respectively). In contrast, an inverse distribution was found among wild bird strains, with 34% of the strains *cgtB*^+^ and only 6% *wlaN*^+^. Therefore, among the LOS^SIAL^ proficient strains, the *wlaN* gene was responsible for the LOS modification in 72% of the human and broiler chicken strains, whereas the *cgtB* gene was responsible in 85% of the wild bird strains.

### 3.2. The Presence of the cgtB and wlaN Genes is Associated with Certain MLST Clonal Complexes

The phylogenetic population structure of the *C. jejuni* collection used in this work has previously been reported [15]. The most frequent clonal complexes (CC) detected were ST-21, ST-1275, ST-45, and ST-257, each CC with 24, 16, 13, and 12 strains, respectively.

The LOS^SIAL^ producing strains grouped within certain CC (Table 2). The most predominant clonal complex, the ST-21 CC, which was only found among human and broiler strains, showed the highest occurrence (78%) of LOS^SIAL^ related genes, with 14 *wlaN*^+^ and 3 *cgtB*^+^ isolates. Remarkably, the three *cgtB*^+^ strains belong to the ST-883. Within the ST-1275 CC, which was only found in wild bird strains, 44% were LOS^SIAL^ (3 *wlaN*^+^ and 4 *cgtB*^+^). It should be highlighted that the only three *wlaN*^+^ strains identified among wild bird isolates belong to this CC.

The ST-45 CC is a multihost complex found among the three populations. However, all LOS^SIAL^ strains from this clonal complex (54%) were isolated only from wild birds and they carry the *cgtB* gene. The strains belonging to the ST-257 CC, isolated from human patients and broiler chickens, were negative for the presence of LOS^SIAL^-related genes. Among the non-predominant CC (containing 3 to 10 strains per CC), the ST-179 showed the highest percentage of LOS^SIAL^ strains (57.5%). This CC was only found among wild bird strains and accordingly, all LOS^SIAL^ strains were *cgtB*^+^. The 37.5% of the ST-607 CC strains, found in humans and broiler chickens, carry LOS^SIAL^-related genes (1 *wlaN*^+^ and 2 *cgtB*^+^). None of the strains belonging to the ST-48, 61, 354, 464, and 952 CC were positive for LOS^SIAL^-related genes.

The fact that within the same sequence type we found strains carrying different LOS determinants is consistent with previous studies, suggesting that the LOS locus is one of the hypervariable regions within the *C. jejuni* genome [20].

### 3.3. Homopolymeric G-tract variants in wlaN and cgtB genes

**Figure 2:**
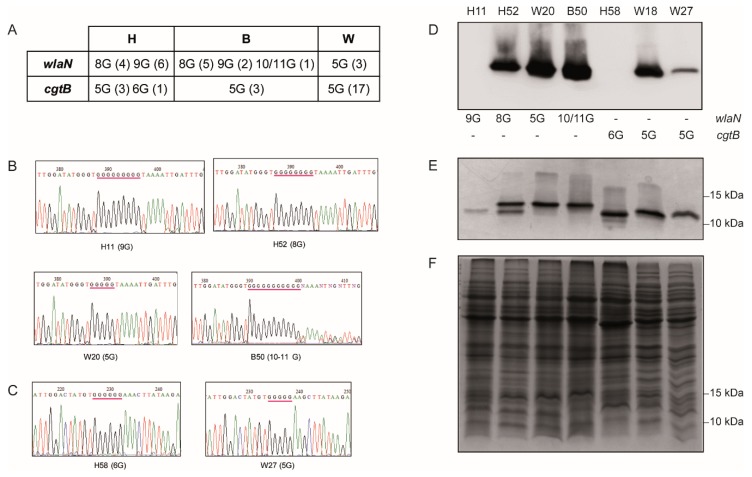
G-tract characterization within the *wlaN* and *cgtB* genes. (**A**) Homopolymeric G-tracts identified in *wlaN* and *cgtB* genes from human (H), broiler chicken (B), and wild bird strains (W). The different homopolymeric variants identified are indicated and the number of strains is shown between parentheses. (**B**) Sequence of the intragenic homopolymeric G-tracts (indicated by red lines) of the *wlaN* gene of the strains H11, H52, W20, and B50. The number of G residues within the G-tract are indicated between parentheses. (**C**) Sequence of the intragenic homopolymeric G-tracts (indicated by red lines) of the *cgtB* gene of the strains H58 and W27. The number of G residues within the G-tracts are indicated between parentheses. (**D**) Detection of LOS^SIAL^ in purified LOS samples from cultures of the indicated strains grown onto CBA plates for 48 h at 42 °C. LOS^SIAL^ was detected by Western blot using HRP-CT. Below, the presence for each strain of either the *wlaN* or *cgtB* gene is indicated and the G-tract variant detected. (**E**) Silver-stained 15% SDS-PAGE of the same samples of purified LOS as in D. (**F**) Coomassie-stained 15% SDS-PAGE of whole cell extracts from the cultures used to obtain the purified LOS samples analyzed in D and E. In E and F, the migration of the 10 and 15 kDa proteins from the molecular mass marker are indicated.

As described earlier, the *wlaN* gene carries an intragenic homopolymeric G-tract [12]. It is assumed that the number of G-residues can vary after DNA replication by a slipped strand mechanism. It has been identified that *wlaN* alleles carry homolopymeric G-tract with different numbers of G residues. So far, G-tracts with 6, 8, and 9G-residues have been described [12,14]. From those, the 8G-tract variant is the only one rendering a full-length product (ON). The homopolymeric G-tract was characterized for all *wlaN^+^* strains of our collection (Figure 2A,B). Variants carrying the previously identified 8 and 9G-tracts were found in both broiler chicken and human isolates. Additionally, new G-tract variants were identified during our study. In the broiler chicken isolate B50, the *wlaN* sequence reveals a mixed population, with 10G- (OFF) and 11G-tracts (ON) (Figure 2B). Strikingly, the three unique *wlaN*^+^ isolates among the wild bird strains (W09, W20, and W25), belonging to the ST-1275 CC, carry a 5G-tract variant, which was not previously described (Table 2). Detection of LOS^SIAL^ in extracts of the W20 strain corroborate that the newly described *wlaN* 5G-tract variant renders expression of a functional β-1,3-galactosyltransferase (Figure 2D). It is worth mentioning that the nucleotide sequence surrounding the G-tract has very high identity among the sequenced *wlaN* variants (Figure S1).

It has not yet been reported whether *cgtB* expression is under phase variation control. Interestingly, the *cgtB* gene carries a G-tract which is located in a different relative position within the coding sequence as compared to *wlaN*. Within the *wlaN* coding sequence, the G-tract is located at position 331 from 912 nt; whereas, within the *cgtB* coding sequence, the newly described G-tract is located at position 476 from 906 nt (Figure S2). In most *cgtB^+^ C. jejuni* sequenced strains, the *cgtB* gene carries a 5G-tract, which renders a full length protein (Table 2, [21]). The length of the homopolymeric G-tract in the *cgtB^+^* isolates was determined. Although most *cgtB^+^* strains carry a 5G-tract (ON), the human isolate H58 carries a 6G-tract, which will generate a truncated protein (Figure 2C). These results suggest that the homopolymeric G-tract which is present within the *cgtB* gene may also be mediating phase variation control. As for *wlaN*, the sequences surrounding the G tract of the *cgtB* variants show very high identity (Figure S3).

**Figure 3:**
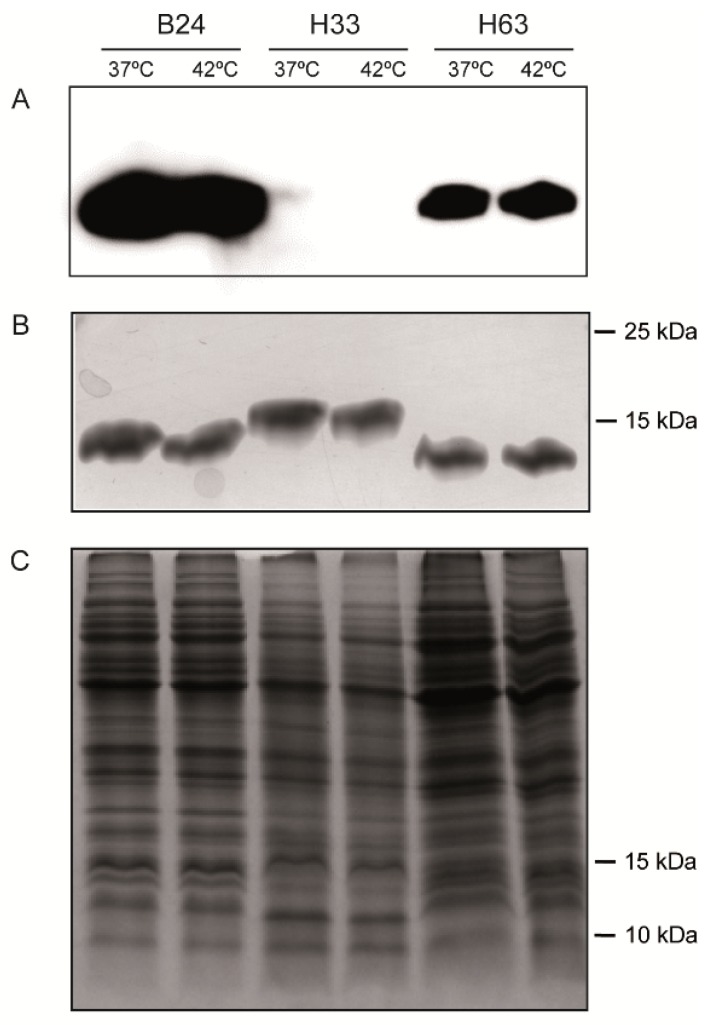
Analysis of LOS from *C. jejuni* strains grown at either 37 °C or 42 °C. (**A**). Detection of LOS^SIAL^ in purified LOS samples from cultures of the indicated strains grown onto CBA plates for 48 h at either 37 °C or 42 °C. LOS^SIAL^ was detected by Western blot using HRP-CT. (**B**). Silver-stained 15% SDS-PAGE of the same samples of purified LOS as in (**A**). (**C**). Coomassie-stained 15% SDS-PAGE of whole cell extracts from the cultures used to obtain the purified LOS samples analyzed in (**A**,**B**). In (**B**,**C**), the migration of the 10 and 15 kDa proteins from the molecular mass marker is indicated.

The phase variation control involves phenotypic diversity (LOS and LOS^SIAL^) within a clonal population. The presence of LOS^SIAL^ on *C. jejuni* surface can be detected by its ability to bind cholera toxin subunit B (CT) [12]. Phenotypic characterization of the LOS^SIAL^ production in *wlaN^+^* and *cgtB^+^* strains was performed. Representative results are shown in Figure 2D and Figure 3. LOS^SIAL^ was detected in all extracts from either *wlaN^+^* or *cgtB^+^* strains carrying “ON” G-tracts and in most of the genotypically “OFF” characterized strains, as in the case of H63 strain (Figure 3). Exceptionally, in extracts from H11 and H58 strains, carrying “OFF” G-tracts were negative for LOS^SIAL^ detection (Figure 2D). LOS silver staining indicates that both strains indeed produce LOS structures, although it does not carry the modification recognized by the cholera toxin (Figure 2E).

### 3.4. The production of LOS^SIAL^ is Not Affected by Temperature

*C. jejuni* colonizes different hosts, including birds as broilers and mammals as humans. The LOS^SIAL^ production at 42 °C and 37 °C, resembling the gastrointestinal tract temperature in broiler chickens and humans, respectively, was monitored. No difference was detected in the amount of LOS in extracts from the LOS^SIAL^ (strains B24 and H63) or non-sialylated LOS (H33) from cultures grown at 42 °C and 37 °C (Figure 3).

### 3.5. Sialylation is Not Needed for Invasiveness

The role of LOS^SIAL^ structures in *C. jejuni* pathogenicity is not fully understood. It was proposed that the LOS^SIAL^ presence on the surface of *C. jejuni* might promote invasion of eukaryotic cells [14,22,23]. However, controversial data is reported by different authors [24,25].

**Figure 4:**
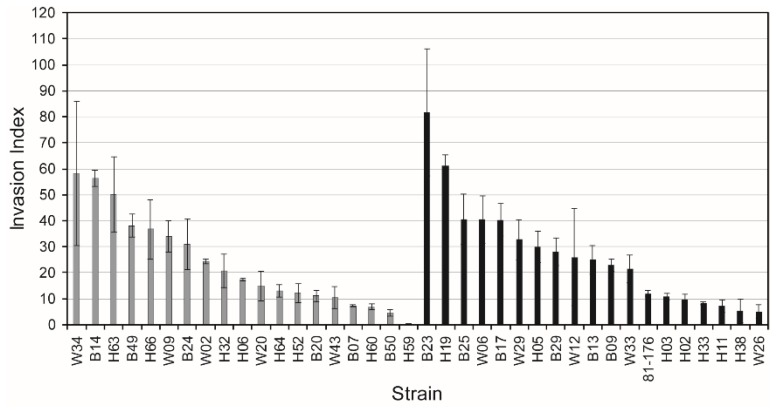
Relation between invasion index and LOS^SIAL^ production in *C. jejuni* strains. The invasion index was calculated as the percentage of intracellular cells from the total cell-associated bacteria after 3 h infection of Caco-2 cells with the indicated strains. The average and standard deviation of three independent experiments are shown. Grey and black bars indicate strains that express or do not express LOS^SIAL^, respectively.

The ability to invade Caco-2 cells by 38 *C. jejuni* strains (37 from our collection and the pathogenic 81-176 strain) was tested. Half of the strains were LOS^SIAL^ proficient (*wlaN*^+^ or *cgtB^+^)*. The invasion index was calculated and the results indicate that no correlation exists between the presence of LOS^SIAL^ structures and invasiveness (Figure 4).

## 4. Discussion

The presence of LOS^SIAL^-related genes—*wlaN* and *cgtB*—coding for β-1,3-glycosyltransferases is correlated with the ability to trigger GBS in patients suffering from campylobacteriosis. In our study, the prevalence of these genes was determined among *C. jejuni* isolates from human patients and broiler chickens since the consumption of undercooked chicken meat is the most common transmission route of *C. jejuni* to humans. *C. jejuni* isolates from wild birds were also analyzed since circulation of *C. jejuni* strains among poultry and wild birds has been reported [26]. Furthermore, wild birds are potential sources of human campylobacteriosis [4,27]. Previous studies focused mostly on comparing human and broiler chicken isolates and no correlation between the origin of the strains and the presence of *wlaN* and *cgtB* genes was found. It has been described that 40% to 60% of human isolates and 28% to 90% of broiler chicken isolates carry LOS^SIAL^ related genes [21,28,29,30,31,32]. Similarly, in our collection, no significant differences were found in the prevalence of LOS^SIAL^-related genes among human and broiler chicken isolates (28% and 22%, respectively). Remarkably, our data indicate that the prevalence of LOS^SIAL^-related genes is higher among the wild bird isolates (40%). 

Among the LOS^SIAL^ proficient strains, a differential distribution of the *cgtB* and *wlaN* genes between the human/broiler chicken isolates and those from wild birds was found. The *wlaN* gene is detected in 72% of the LOS^SIAL^ strains from humans and broiler chickens, whereas the most prevalent LOS^SIAL^-related gene in wild bird isolates is *cgtB* (85%). The information available on the prevalence of LOS^SIAL^-related genes among wild bird isolates is limited. No previous data exist on the prevalence of the *cgtB* gene, and the presence of the *wlaN* gene has been estimated between 11% and 17% of wild bird isolates [33,34]. Although several reports indicated that *cgtB* and *wlaN* may coexist [14,31], our data suggest the contrary, since none of the isolates carry both genes.

A search for the presence of LOS^SIAL^-related genes among 136 *C. jejuni* genome sequences available in the NCBI database was performed (Table S1). In agreement with our data, the presence of LOS^SIAL^-related genes was confirmed in 40% of the strains, with *wlaN* and *cgtB* present in 22% and 18%, respectively. It should be noted that 87% of LOS^SIAL^ positive strains were isolated from human or broiler chicken hosts and the remaining 13% from other domestic animals. Moreover, none of the genome sequences were found to simultaneously carry *wlaN* and *cgtB*. 

The *wlaN* gene is under phase variation control [12], meaning that within a clonal population, cells can express sialylated and unsialylated LOS. This phenomenon is achieved since the number of G residues in a G-tract within the *wlaN* coding sequence can be randomly altered during replication, rendering a truncated or a fully functional protein. Three variants were previously described (6G, 8G, and 9G) [12,13]. Here, we revealed three new variants: a 5G-tract found in three wild bird isolates and 10- and 11G-tracts, which were both found in a broiler chicken isolate (Figure 2B and Figure S1). Remarkably, the *cgtB* gene carries a G-tract within the coding sequence, but a potential phase variation regulation was not reported. Here, two *cgtB* variants were found (Figure 2C, Figure S3): a 5G-tract, detected in most *cgtB*^+^ strains, rendering a full protein and a 6G-tract, detected only in one isolate, rendering a truncated peptide. Interestingly, the genomic sequence of the well-characterized strain 81–176 indicates the presence of a truncated *cgtB* gene with a 6G-tract. Overall, our data suggest that, similar to the *wlaN* gene, *cgtB* is under phase variation regulation by altering the number of residues in the G-tract. Further studies will be required to fully characterize the mechanism behind the described phenomenon. 

Despite the well-established role of the *cgtB* and *wlaN* genes in triggering GBS [6], the relevance of these genes in the pathogenesis of *C. jejuni* during gastrointestinal infection remains unclear. The lack of thermoregulation of LOS and/or LOS^SIAL^ suggests that its production is neither promoted nor repressed in a specific host temperature. Some authors reported a link between LOS sialylation and the ability to invade eukaryotic cells [14,22,23]. In agreement with other reports [24,25], our data suggest that no correlation exists between these two processes since no differences in the invasion index were detected between LOS^SIAL^ and non-sialylated strains.

Overall, our data reveal a closer relationship between human and broiler chicken isolates as compared to wild bird isolates, which is in agreement with the previous characterization of the strain collection in terms of population structure, drug resistance, and virulence factor profiling [15]. The differential distribution of *wlaN* and *cgtB* genes may also indicate a host-dependent distribution of the LOS^SIAL^-related genes, with *wlaN* positively selected among broiler chickens and consequently, also among human isolates, and *cgtB* positively selected among wild birds.

## Figures and Tables

**Table 1:** Primers used in this work.

Primer	Sequence 5’–3’	PCR product (bp)	Reference
cgtB-F	TTAAGAGCAAGATATGAAGGT	740	[12]
cgtB-R	GCACATAGAGAACGCTACAA		
wlaN-F	TGCTGGGTATACAAAGGTTGTG	561	[16]
wlaN-R	AGGTCCATTACCGCATACCA		
gltA-F	GCCCAAAGCCCATCAAGCGGA	141	[17]
gltA-R	GCGCTTTGGGGTCATGCACA		

**Table 2:** Collection of *C. jejuni* isolated from human patients (H), broiler chikens (B), and wild birds (W). Strains are organized attending to their ST Clonal Complexes (ST CC) and sequence types (ST). S means a singlenton ST. *cgtB* and *wlaN* positive strains are indicated (black squares) and the number of G residues found in the G-tract is indicated.

Strain	*cgtB*	*wlaN*	ST-CC (ST)	Strain	*cgtB*	*wlaN*	ST-CC (ST)	Strain	*cgtB*	*wlaN*	ST-CC (ST)
H01			S (441)	H62			ST-206 (572)	B45			ST-354 (354)
H72			S (441)	H68			ST-21 (19)	W28			ST-354 (354)
B44			S (441)	H06		9	ST-21 (19)	W54			ST-354 (354)
H53			S (531)	H11		9	ST-21 (21)	W55			ST-354 (354)
B16			S (531)	H32		9	ST-21 (21)	W56			ST-354 (354)
W02	5		S (996)	H52		8	ST-21 (21)	B37			ST-354 (8498)
W11			S (1261)	H59		8	ST-21 (21)	H61	5		ST-42 (459)
W13			S (1343)	B20		9	ST-21 (21)	H58	6		ST-42 (4016)
W23			S (1343)	B36		8	ST-21 (21)	H46			ST-443 (51)
H07			S (1710)	B46		8	ST-21 (21)	H19			ST-443 (5799)
B10			S (1710)	H12			ST-21 (50)	W12			ST-446 (3552)
B26			S (1710)	H34		9	ST-21 (50)	H48			ST-45 (45)
B35			S (1710)	H35			ST-21 (50)	B27			ST-45 (45)
B02			S (2331)	H66		8	ST-21 (50)	B31			ST-45 (45)
W17	5		S (2351)	H73		9	ST-21 (50)	W30			ST-45 (45)
W06			S (4355)	B07		8	ST-21 (50)	W32	5		ST-45 (45)
W07			S (4355)	B14		8	ST-21 (50)	W36	5		ST-45 (45)
W08			S (4355)	B50		10/11	ST-21 (50)	W37	5		ST-45 (45)
B39			S (7114)	H37	5		ST-21 (883)	W40	5		ST-45 (45)
H33			S (8479)	H60	5		ST-21 (883)	W43	5		ST-45 (45)
W53			S (8514)	B06	5		ST-21 (883)	W44	5		ST-45 (45)
W05			ST-1034 (4001)	B17			ST-21 (883)	B42			ST-45 (137)
W24			ST-1275 (637)	H54			ST-21 (1214)	B22			ST-45 (652)
W27	5		ST-1275 (637)	H08			ST-21 (3769)	W47	5		ST-45 (8512)
W03			ST-1275 (1223)	H02			ST-21 (4664)	H18			ST-464 (464)
W10	5		ST-1275 (1223)	H03			ST-257 (257)	B08			ST-464 (464)
W15	5		ST-1275 (1223)	H50			ST-257 (257)	B15			ST-464 (464)
W18	5		ST-1275 (1223)	H56			ST-257 (257)	H38			ST-48 (48)
W21			ST-1275 (1223)	B52			ST-257 (367)	B09			ST-48 (48)
W22			ST-1275 (1268)	B53			ST-257 (367)	W29			ST-48 (48)
W04			ST-1275 (1275)	B54			ST-257 (367)	H71			ST-49 (49)
W09		5	ST-1275 (1275)	B55			ST-257 (367)	H51			ST-52 (52)
W20		5	ST-1275 (1275)	B56			ST-257 (367)	B21			ST-574 (305)
W14			ST-1275 (1292)	B57			ST-257 (367)	B25			ST-574 (305)
W16			ST-1275 (1292)	H67			ST-257 (2254)	B04	5		ST-607 (607)
W26			ST-1275 (3049)	H69			ST-257 (2254)	B41	5		ST-607 (607)
W19			ST-1275 (3629)	B13			ST-257 (2254)	H04			ST-607 (904)
W25		5	ST-1275 (8511)	B33			ST-283 (267)	B38			ST-607 (904)
W33			ST-179 (179)	B28			ST-353 (5)	B47			ST-607 (904)
W41			ST-179 (220)	B29			ST-353 (5)	B24		8	ST-607 (1707)
W34	5		ST-179 (2209)	H63		9	ST-353 (353)	B19			ST-607 (7110)
W39	5		ST-179 (2209)	B48			ST-353 (356)	B51			ST-607 (7110)
W46	5		ST-179 (2209)	H36			ST-353 (400)	H05			ST-61 (61)
W48			ST-179 (2209)	B05			ST-353 (400)	H40			ST-61 (61)
W49	5		ST-179 (2209)	B18			ST-353 (400)	H57			ST-61 (61)
B49		9	ST-206 (46)	B30			ST-353 (400)	H65			ST-61 (61)
H64		8	ST-206 (227)	B40			ST-353 (400)	H70			ST-61 (61)
H09			ST-206 (572)	H49			ST-354 (354)	W50			ST-952 (8513)
H13			ST-206 (572)	H74			ST-354 (354)	W51			ST-952 (8513)
H14			ST-206 (572)	B23			ST-354 (354)	W52			ST-952 (8513)

## Supplementary Materials

The following are available online at https://www.mdpi.com/2076-2607/8/3/325/s1, Figure S1: *wlaN* nucleotide sequence surrounding the position of G-tract in the *wlaN* positive strains, Figure S2: Alignment of *wlaN* and *cgtB* nucleotide sequences, Figure S3: *cgtB* nucleotide sequence surrounding the position of the G-tract in the *cgtB* positive strains, Table S1: Presence of the *wlaN* and *cgtB* genes in complete genome sequences of *C. jejuni* from the NCBI database.
