# Supplementary material for: Differential Distribution of the wlaN and cgtB Genes, Associated with Guillain-Barré Syndrome, in Campylobacter jejuni Isolates from Humans, Broiler Chickens, and Wild Birds
Source: Microorganisms. 2020 Feb 26;8(3):325. doi: 10.3390/microorganisms8030325 (PMC7142995; doi:10.3390/microorganisms8030325)
Supplement: Supplementary file 1 [file microorganisms-08-00325-s001.zip › Table S1.pdf]

Table S1. Presence of *wlaN* and *cgtB* genes determined in the complete genome sequences from *C. jejuni* available in the NCBI database (13th May 2018), by homology searching with specific primers for LOS classes A, B and C described previously (Parker et al. Comparison of *Campylobacter jejuni* Lipooligosaccharide Biosynthesis Loci from a Variety of Sources. J Clin Microbiol 2005;43:2771–81).

**Strain**	**Accession number**	***wlaN***	***cgtB***	**origin**	**ST-CC (ST)**
100	CP023446.1	+		chicken	nd
14980A	NZ_CP017029.1/CP017029.1		+	turkey	S (1839)
269.97	CP000768.1			human	S (1845)
MTVDSCj16	NZ_CP017033.1/CP017033.1		+	chicken	S (1911)
T1-21	NZ_CP013116.1/CP013116.1			chicken	S (3579)
F38011	NZ_CP006851.1/CP006851.1			human	S (3644)
414	CM000855.1			nd	S (3704)
35925	NZ_CP010906.1/CP010906.1			human	S (5843)
NCTC12662	NZ_CP019965.1/CP019965.1			nd	S (5843)
ATCC 35925	NZ_CP020045.1/CP020045.1			pigeon	S (5843)
1336	NZ_CM000854.1/CM000854.1			nd	S (841)
CFSAN032806	NZ_CP023543.1/CP023543.1	+		chicken	ST- 206 (222)
00-2544	NC_022353.2/CP006709.2	+		human	ST-21 (21)
00-2538	NC_022351.2/CP006707.2	+		human	ST-21 (21)
00-2426	NC_022352.2/CP006708.2	+		human	ST-21 (21)
00-2425	NC_022362.2/CP006729.2	+		human	ST-21 (21)
NS4-1-1	NZ_CP007191.1/CP007191.1	+		chicken	ST-21 (21)
NS4-5-1	NZ_CP007192.1/CP007192.1	+		chicken	ST-21 (21)
NS4-9-1	NZ_CP007193.1/CP007193.1	+		chicken	ST-21 (21)
81-176_G1_B7	CP022551.1		+	human	ST-21 (21)
D42a	CP007751.1	+		chicken	ST-21 (21)
NCTC 11168-Kf1	NZ_CP006686.1/CP006686.1	+		human	ST21 (43)
NCTC 11168	NC_002163.1/AL111168.1	+		human	ST-21 (43)
NCTC 11168-BN148	INC_018521.1/HE978252.1	+		human	ST-21 (43)
NCTC 11168-K12E5	NZ_CP006685.1/CP006685.1	+		human	ST-21 (43)
NCTC 11168-mcK12E5	NZ_CP006687.1/CP006687.1	+		mice	ST-21 (43)
NCTC 11168-mfK12E5	NZ_CP006688.1/CP006688.1			human	ST-21 (43)
NCTC 11168-rNRC	NZ_CP006689.1/CP006689.1	+		human	ST-21 (43)
FDAARGOS_263	NZ_CP022077.1/CP022077.1	+		human	ST-21 (43)

11168H/lacY	NZ_CP022439.1/CP022439.1	+		human	ST-21 (43)
11168H/araE	NZ_CP022559.1/CP022559.1	+		human	ST-21 (43)
PT14	NC_018709.4/CP003871.4	+		human	ST-21 (50)
RM1285	NZ_CP012696.1/CP012696.1	+		chicken	ST-21 (50)
OD267	NZ_CP014744.1/CP014744.1	+		chicken	ST-21 (50)
WP2202	NZ_CP014742.1/CP014742.1	+		chicken	ST-21 (50)
IA3902	NC_017279.1/CP001876.1	+		ovine	ST-21 (8)
01-1512	NZ_CP010072.1/CP010072.1	+		human	ST-21 (8)
00-0949	NZ_CP010301.1/CP010301.1	+		human	ST-21 (8)
00-6200	NZ_CP010307.1/CP010307.1		+	human	ST-21 (806)
YH001	CP010058.1			beef	ST-21 (806)
HF5-4A-4	NZ_CP007188.1/CP007188.1		+	chicken	ST-21 (861)
MTVDSCj07	NZ_CP017031.1/CP017031.1	+		chicken	ST-21 (8789)
FDAARGOS_422	NZ_CP023867.1/CP023867.1		+	human	ST-21 (883)
YH002	NZ_CP020776.1/CP020776.1	+		calf	ST-21 (982)
RM1285	NZ_CP015209.1/CP015209.1		+	chicken	ST-22 (22)
RM3420	NZ_CP017456.1/CP017456.1		+	human	ST-22 (22)
FORC_046	NZ_CP017229.1/CP017229.1		+	human	ST-22 (22)
81116 NCTC 11828	NC_009839.1/CP000814.1			human	ST-283 (267)
00-1597	NZ_CP010306.1/CP010306.1		+	human	ST-353 (2132)
M129	NZ_CP007749.1/CP007749.1		+	human	ST-353 (353)
R14	CP005081.1			nd	ST-353 (356)
IF1100	NZ_CP017863.1/CP017863.1			chicken	ST-353 (462)
RM1221	NC_003912.7/CP000025.1			chicken	ST-354 (354)
S3	NC_017281.1/CP001960.1			chicken	ST-354 (354)
FDAARGOS_421	NZ_CP023866.1/CP023866.1			chicken	ST-354 (354)
ICDCCJ07001	CP002029.1		+	human	ST-362 (2993)
RM3197	NZ_CP012689.1/CP012689.1		+	human	ST-362 (362)
RM3196	NZ_CP012690.1/CP012690.1		+	human	ST-362 (362)
NCTC11351	NZ_LN831025.1/LN831025.1		+	nd	ST-403 (403)
FDAARGOS_262	NZ_CP022076.1/CP022076.1		+	bovine	ST-403 (403)
81-176_G1_B0	NZ_CP022440.1/CP022440.1		+	human	ST-42 (604)
81-176	NC_008787.1/CP000538.1		+	human	ST-42 (604)
M1	NC_017280.1/CP001900.1			human	ST-45 (137)
CJM1cam	NZ_CP012149.1/CP012149.1			human	ST-45 (137)
4031	INC_022529.1/HG428754.1			water	ST-45 (45)
HF5-5-1	NZ_CP007189.1/CP007189.1			farm	ST-45 (45)
HF5-7-1	NZ_CP007190.1/CP007190.1			farm	ST-45 (45)
RM1246-ERRC	NZ_CP022470.1/CP022470.1			human	ST-45 (45)
FDAARGOS_266	NZ_CP022080.1/CP022080.1			nd	ST-45 (583)
MTVDSCj20	NZ_CP008787.1/CP008787.1			chicken	ST-45 (8785)

RM3194	NZ_CP014344.1/CP014344.1		+	human	ST-460 (1471)
MTVDSCj13	NZ_CP017032.1/CP017032.1		+	chicken	ST-460 (460)
32488	NC_021834.1/CP006006.1		+	human	ST-48 (1460)
FDAARGOS_265	NZ_CP022079.1/CP022079.1		+	human	ST-48 (48)
CG8421	NZ_CP005388.1/CP005388.1			human	ST-52 (1919)
FJ3124	NZ_CP017862.1/CP017862.1			chicken	ST-52 (3642)
YQ2210	NZ_CP017859.1/CP017859.1	+		turkey	ST-607 (1212)
ZP3204	NZ_CP017856.1/CP017856.1	+		chicken	ST-607 (1212)
TS1218	NZ_CP017860.1/CP017860.1		+	chicken	ST-607 (607)
104	CP023343.1		+	chicken	ST-607 (607)
CJ677CC519	NZ_CP010471.1/CP010471.1			human	ST-677 (677)
CJ677CC002	NZ_CP010472.1/CP010472.1			human	ST-677 (677)
CJ677CC534	NZ_CP010473.1/CP010473.1			human	ST-677 (677)
CJ677CC536	NZ_CP010474.1/CP010474.1			human	ST-677 (677)
CJ677CC521	NZ_CP010476.1/CP010476.1			human	ST-677 (677)
CJ677CC526	NZ_CP010477.1/CP010477.1			human	ST-677 (677)
CJ677CC036	NZ_CP010479.1/CP010479.1			human	ST-677 (677)
CJ677CC524	NZ_CP010480.1/CP010480.1			human	ST-677 (677)
CJ677CC016	NZ_CP010481.1/CP010481.1			human	ST-677 (677)
CJ677CC535	NZ_CP010483.1/CP010483.1			human	ST-677 (677)
CJ677CC092	NZ_CP010488.1/CP010488.1			human	ST-677 (677)
CJ677CC530	NZ_CP010489.1/CP010489.1			human	ST-677 (677)
CJ677CC532	NZ_CP010490.1/CP010490.1			human	ST-677 (677)
CJ677CC529	NZ_CP010491.1/CP010491.1			human	ST-677 (677)
CJ677CC531	NZ_CP010492.1/CP010492.1			human	ST-677 (677)
CJ677CC062	NZ_CP010493.1/CP010493.1			human	ST-677 (677)
CJ677CC059	NZ_CP010494.1/CP010494.1			human	ST-677 (677)
CJ677CC537	NZ_CP010498.1/CP010498.1			human	ST-677 (677)
CJ677CC528	NZ_CP010500.1/CP010500.1			human	ST-677 (677)
CJ677CC538	NZ_CP010495.1/CP010495.1			human	ST-677 (677)
CJ677CC520	NZ_CP010501.1/CP010501.1			human	ST-677 (677)
CJ677CC014	NZ_CP010502.1/CP010502.1			human	ST-677 (677)
CJ677CC039	NZ_CP010503.1/CP010503.1			human	ST-677 (677)
CJ677CC527	NZ_CP010506.1/CP010506.1			human	ST-677 (677)
CJ677CC078	NZ_CP010507.1/CP010507.1			human	ST-677 (677)
CJ677CC523	NZ_CP010508.1/CP010508.1			human	ST-677 (677)
CJ677CC040	NZ_CP010510.1/CP010510.1			human	ST-677 (677)
CJ677CC061	NZ_CP010511.1/CP010511.1			human	ST-677 (677)
CJ677CC533	NZ_CP010458.1/CP010458.1			human	ST-677 (677)
CJ677CC047	NZ_CP010459.1/CP010459.1			human	ST-677 (677)
CJ677CC058	NZ_CP010460.1/CP010460.1			human	ST-677 (677)

CJ677CC013	NZ_CP010461.1/CP010461.1			human	ST-677 (677)
CJ677CC100	NZ_CP010462.1/CP010462.1			human	ST-677 (677)
CJ677CC522	NZ_CP010463.1/CP010463.1			human	ST-677 (677)
CJ677CC094	NZ_CP010464.1/CP010464.1			human	ST-677 (677)
CJ677CC008	NZ_CP010465.1/CP010465.1			human	ST-677 (677)
CJ677CC024	NZ_CP010467.1/CP010467.1			human	ST-677 (677)
CJ677CC064	NZ_CP010468.1/CP010468.1			human	ST-677 (677)
CJ677CC525	NZ_CP010469.1/CP010469.1			human	ST-677 (677)
CJ677CC026	NZ_CP010470.1/CP010470.1			human	ST-677 (677)
CJ677CC086	NZ_CP010485.1/CP010485.1			human	ST-677 (677)
CJ677CC095	NZ_CP010486.1/CP010486.1			human	ST-677 (677)
CJ677CC010	CP010478.1			human	ST-677 (677)
CJ677CC073	NZ_CP010475.1/CP010475.1			human	ST-677 (794)
CJ677CC041	NZ_CP010482.1/CP010482.1			human	ST-677 (794)
CJ677CC032	NZ_CP010496.1/CP010496.1			human	ST-677 (794)
CJ677CC033	NZ_CP010497.1/CP010497.1			human	ST-677 (794)
CJ677CC542	NZ_CP010499.1/CP010499.1			human	ST-677 (794)
CJ677CC085	NZ_CP010504.1/CP010504.1			human	ST-677 (794)
CJ677CC052	NZ_CP010505.1/CP010505.1			human	ST-677 (794)
CJ677CC540	NZ_CP010509.1/CP010509.1			human	ST-677 (794)
CJ677CC539	NZ_CP010457.1/CP010457.1			human	ST-677 (794)
CJ677CC541	NZ_CP010466.1/CP010466.1			human	ST-677 (794)
CJ677CC034	NZ_CP010484.1/CP010484.1			human	ST-677 (794)
CJ677CC012	NZ_CP010487.1/CP010487.1			human	ST-677 (794)
