# Supplementary material for: Differential Distribution of the wlaN and cgtB Genes, Associated with Guillain-Barré Syndrome, in Campylobacter jejuni Isolates from Humans, Broiler Chickens, and Wild Birds
Source: Microorganisms. 2020 Feb 26;8(3):325. doi: 10.3390/microorganisms8030325 (PMC7142995; doi:10.3390/microorganisms8030325)
Supplement: Supplementary file 1 [file microorganisms-08-00325-s001.zip › FIG S2.pdf]

```
wlaN       ATGAGTCAAATTTCCATCATACTACCAACTTATAATGTGGAAAAATATATTGCTAGAGCA      60
cgtB       ATGTTTAAAATTTCAATCATCTTACCAACTTATAATGTGGAACAATATATAGCAAGGGCA      60
           ***: *.*******.*****. ********************.*******:**:**.***

wlaN       TTAGAAAGTTGCATTAACCAAACTTTTAAAGATATAGAAATCATTGTAGTAGATGATTGT      120
cgtB       ATAGAAAGCTGTATCAATCAGACTTTTAAAGATATAGAAATAATTGTAGTTGATGATTGT      120
           :******* ** ** ** **.*******************.********:*********

wlaN       GGTAATGATAAAAGTATAGATATAGCTAAAGAGTATGCTAGTAAAGATGATAGAATAAAA      180
cgtB       GGAAATGATAATAGTATAAATATAGCCAAAGAATACTCTAAAAAAGACAAAAGAATAAAA      180
           **:********:******.******* *****.**  ***.:***** .*:*********

wlaN       ATCATACATAATGAAGAGAATTTAAAGCTTTTAAGAGCAAGATATGAAGGTGCTAAAGTA      240
cgtB       ATAATCCACAATGAAAAAAAACTTAGGTCTTTTAAGAGCAAGATATGAAGGTGTGAAAGTA      240
           **.**.** ******.*.** ***.. *************************  ******

wlaN       GCAACTTCACCTTATATCATGTTTTTAGATTCTGATGATTATTTAGAACTTAATGCTTGC      300
cgtB       GCAAACTCTCCTTATATAATGTTTTTAGATCCTGATGATTATTTGGAACTAAATGCTTGT      300
           ****. **:********.************ *************.*****:********

wlaN       GAAGAATGTATTAAAATTTTGGATATGGGTGGGGGGGGTAAAATTGATTTGTTGTGTTTT      360
cgtB       GAAGAGTGTATAAAAAATTTAGATGAAC------AGGATGAAGTTGATTTAGTGTTTTTC      354
           *****.*****:********.***.:.       .**.*.**.*******. *** ***

wlaN       GAAGCTTTTATTACCAATGCAAAAAAAATCAATAAAAAAAT-TAAATATAAAACAAGGAAA      419
cgtB       AATGCTATTGTTGAAAGTAATGTTATTTCATATAAAAAGTTTGACTTTAATTC-TGGTTT      413
           .*:***:**.**...*.*..:.::*::***:::*****.* *.*.*:***::* :**:::

wlaN       ATACAACAACAAAGAATTTACAATGCAAATACTTAAAAC---TAAAAATCCATTTTGGAC      476
cgtB       TTATAGCAAAAAAGAGTTTGT---GAAAAAAATTATTGCAAAGAAAAATTTATATTGGAC      470
           :** *.***.*****.***.    *.***:*.***::.*    ******  **:******

wlaN       AATGTGGGCTAAAATAATCAAAAAAAGATATTTATTTAAAAGCCTTCAACATGTTAAATCT      536
cgtB       TATGTGGGGGAAACTTATAAGAAAGAAATTGTATTTAGAAGCTTTTGCGAGTTTAAGACT      530
           :*******  ***.*:**.*.***..*::* ******.**** **  .. *  ****.:**

wlaN       CAAAAAAGAAATCAAAATAAATATGGCAGAAGATGCCTTATTATATTATCCTTTGACAAT      596
cgtB       CGAGAAAGATGTTAAAATCAATATGGCTGAAGATGTATTGTTATATTATCCAATGTTAAG      590
           *.*.*****:.* *****.********:******* .**.***********::**: **

wlaN       ATTATCTAATGAAA-------------------TATTTTACTTAACACAACCTTTGTATA      637
cgtB       TCAAGCTCAAAAAAATAGCATATATGAACTGTAATTTATATCATTACGTGCCTAATAATAA      650
           : :* **.*:.***                   *:*:*::*:*:**. ..* ::*.:::*

wlaN       CCCAGCATGTAAATAGCAATTCTATAA---------CAAAT---AATATTAATTCTTTAGA      686
cgtB       TTCAAT-------TTGTAATACTAAGAATGAAGTGCTTGTTAAAAATAATATTCAAGAGT      703
             **.         *:* ***:***:.*         *::.*   **:::::****:: **:

wlaN       AGCTAATATTCAAGAACATAAAATTGTTTTAAATGTTTTAAAATCAATTAAAAATAAAAA      746
cgtB       TGCAGTTGGTTTTAAACTATTTAAGGCA-AAATTATATTTTAAACAAGTAT------TGT      756
           :**:.:*. * ::.***:::::*: * : :**:*.*:**::**:*** **:      :.:

wlaN       AAC-ACCTCTATATTTTCTAATTATATATTTATTAAAAATTCAATTATTG----AAATAT      801
cgtB       AGCGTTCTCT--ATGTGCTAATTAAATATTTGCTATATATTCAAATATATAAAATAAAAA      814
           *.* : ****  ** * *******:******. **:*:******:***:     :**:*:

wlaN       GAACAAAATTTTAATAAAAGAAATATAAATCTTATTTATTATAAAATAAATATTTTATAT      861
cgtB       GAACAAAATTAAT-----GGT-------TACATTGTTAG-CTAAAATAAATAT--TTTAA      859
           **********:::     .*:       ::*:*: ***  .************  *:**:

wlaN       CAAAAAT--ATCAATTCAAATGGAAAAAAATTTTTATATAATTTAATTCCGTAA--- 912
cgtB       CTTTAAAAATTTTATTTAAATATAAAAAATTT---------TTAAAACAATGTTAA 906
           *:::**:  :* :*** ****. *********         ****::*..*.:
```

Figure S2
