# Supplementary material for: Differential Distribution of the wlaN and cgtB Genes, Associated with Guillain-Barré Syndrome, in Campylobacter jejuni Isolates from Humans, Broiler Chickens, and Wild Birds
Source: Microorganisms. 2020 Feb 26;8(3):325. doi: 10.3390/microorganisms8030325 (PMC7142995; doi:10.3390/microorganisms8030325)
Supplement: Supplementary file 1 [file FIG S1.pdf › FIG S1.pdf]

```
H06   ACTTCACCTTATATCATGTTTTTAGATTCTGATGATTATTTAGAACTTAATGCTTGCGAA  60
H11   ACTTCACCTTATATCATGTTTTTAGATTCTGATGATTATTTAGAACTTAATGCTTGCGAA  60
H32   ACTTCACCTTATATCATGTTTTTAGATTCTGATGATTATTTAGAACTTAATGCTTGCGAA  60
H34   ACTTCACCTTATATCATGTTTTTAGATTCTGATGATTATTTAGAACTTAATGCTTGCGAA  60
H63   ACTTCACCTTATATCATGTTTTTAGATTCTGATGATTATTTAGAACTTAATGCTTGCGAA  60
H73   ACTTCACCTTATATCATGTTTTTAGATTCTGATGATTATTTAGAACTTAATGCTTGCGAA  60
B20   ACTTCACCTTATATCATGTTTTTAGATTCTGATGATTATTTAGAACTTAATGCTTGCGAA  60
B49   ACTTCACCTTATATCATGTTTTTAGATTCTGATGATTATTTAGAACTTAATGCTTGCGAA  60
B50   ACTTCACCTTATATCATGTTTTTAGATTCTGATGATTATTTAGAACTTAATGCTTGCGAA  60
H52   ACTTCACCTTATATCATGTTTTTAGATTCTGATGATTATTTAGAACTTAATGCTTGCGAA  60
H59   ACTTCACCTTATATCATGTTTTTAGATTCTGATGATTATTTAGAACTTAATGCTTGCGAA  60
H64   ACTTCACCTTATATCATGTTTTTAGATTCTGATGATTATTTAGAACTTAATGCTTGCGAA  60
H66   ACTTCACCTTATATCATGTTTTTAGATTCTGATGATTATTTAGAACTTAATGCTTGCGAA  60
B07   ACTTCACCTTATATCATGTTTTTAGATTCTGATGATTATTTAGAACTTAATGCTTGCGAA  60
B14   ACTTCACCTTATATCATGTTTTTAGATTCTGATGATTATTTAGAACTTAATGCTTGCGAA  60
B24   ACTTCACCTTATATCATGTTTTTAGATTCTGATGATTATTTAGAACTTAATGCTTGCGAA  60
B36   ACTTCACCTTATATCATGTTTTTAGATTCTGATGATTATTTAGAACTTAATGCTTGCGAA  60
B46   ACTTCACCTTATATCATGTTTTTAGATTCTGATGATTATTTAGAACTTAATGCTTGCGAA  60
W09   ACTTCACCTTATATCATGTTTTTGGATTCTGATGATTATTTAGAACTTAATGCTTGCGAA  60
W20   ACTTCACCTTATATCATGTTTTTGGATTCTGATGATTATTTAGAACTTAATGCTTGCGAA  60
W25   ACTTCACCTTATATCATGTTTTTAGATTCTGATGATTATTTAGAACTTAATGCTTGCGAA  60
      *********************** ************************************

H06   GAATGTATTAAAATTTTGGATATGGGTGGGG--GGGGGTAAAATTGATTTGTTGTGTTTT  118
H11   GAATGTATTAAAATTTTGGATATGGGTGGGG--GGGGGTAAAATTGATTTGTTGTGTTTT  118
H32   GAATGTATTAAAATTTTGGATATGGGTGGGG--GGGGGTAAAATTGATTTGTTGTGTTTT  118
H34   GAATGTATTAAAATTTTGGATATGGGTGGGG--GGGGGTAAAATTGATTTGTTGTGTTTT  118
H63   GAATGTATTAAAATTTTGGATATGGGTGGGG--GGGGGTAAAATTGATTTGTTGTGTTTT  118
H73   GAATGTATTAAAATTTTGGATATGGGTGGGG--GGGGGTAAAATTGATTTGTTGTGTTTT  118
B20   GAATGTATTAAAATTTTGGATATGGGTGGGG--GGGG TAAAATTGATTTGTTGTGTTTT  118
B49   GAATGTATTAAAATTTTGGATATGGGTGGGG--GGGGGTAAAATTGATTTGTTGTGTTTT  118
B50   GAATGTATTAAAATTTTGGATATGGGTGGGGGGGGGGGTAAAATTGATTTGTTGTGTTTT  120
H52   GAATGTATTAAAATTTTGGATATGGGTGGG---GGGGGTAAAATTGATTTGTTGTGTTTT  117
H59   GAATGTATTAAAATTTTGGATATGGGTGGG---GGGGGTAAAATTGATTTGTTGTGTTTT  117
H64   GAATGTATTAAAATTTTGGATATGGGTGGG---GGGGGTAAAATTGATTTGTTGTGTTTT  117
H66   GAATGTATTAAAATTTTGGATATGGGTGGG---GGGGGTAAAATTGATTTGTTGTGTTTT  117
B07   GAATGTATTAAAATTTTGGATATGGGTGGG---GGGGGTAAAATTGATTTGTTGTGTTTT  117
B14   GAATGTATTAAAATTTTGGATATGGGTGGG---GGGGGTAAAATTGATTTGTTGTGTTTT  117
B24   GAATGTATTAAAATTTTGGATATGGGTGGG---GGGGGTAAAATTGATTTGTTGTGTTTT  117
B36   GAATGTATTAAAATTTTGGATATGGGTGGG---GGGGGTAAAATTGATTTGTTGTGTTTT  117
B46   GAATGTATTAAAATTTTGGATATGGGTGGG---GGGGGTAAAATTGATTTGTTGTGTTTT  117
W09   GAATGTATTAAAATTTTGGATATGGGT------GGGGGTAAAATTGATTTGTTGTGTTTT  114
W20   GAATGTATTAAAATTTTGGATATGGGT------GGGGGTAAAATTGATTTGTTGTGTTTT  114
W25   GAATGTATTAAAATTTTGGATATGGGT------GGGGGTAAAATTGATTTGTTGTGTTTT  114
      ***************************      ***************************

H06   GAAGCTTTTATTACCAATGCAAAAAAATCAATAAAAAAATTAAATATAAAACAAGGAAAA  178
H11   GAAGCTTTTATTACCAATGCAAAAAAATCAATAAAAAAATTAAATATAAAACAAGGAAAA  178
H32   GAAGCTTTTATTACCAATGCAAAAAAATCAATAAAAAAATTAAATATAAAACAAGGAAAA  178
H34   GAAGCTTTTATTACCAATGCAAAAAAATCAATAAAAAAATTAAATATAAAACAAGGAAAA  178
H63   GAAGCTTTTATTACCAATGCAAAAAAATCAATAAAAAAATTAAATATAAAACAAGGAAAA  178
H73   GAAGCTTTTATTACCAATGCAAAAAAATCAATAAAAAAATTAAATATAAAACAAGGAAAA  178
B20   GAAGCTTTTATTACCAATGCAAAAAAATCAATAAAAAAATTAAATATAAAACAAGGAAAA  178
B49   GAAGCTTTTATTACCAATGCAAAAAAATCAATAAAAAAATTAAATATAAAACAAGGAAAA  178
B50   GAAGCTTTTATTACCAATGCAAAAAAATCAATAAAAAAATTAAATATAAAACAAGGAAAA  180
H52   GAAGCTTTTATTACCAATGCAAAAAAATCAATAAAAAAATTAAATATAAAACAAGGAAAA  177
H59   GAAGCTTTTATTACCAATGCAAAAAAATCAATAAAAAAATTAAATATAAAACAAGGAAAA  177
H64   GAAGCTTTTATTACCAATGCAAAAAAATCAATAAAAAAATTAAATATAAAACAAGGAAAA  177
H66   GAAGCTTTTATTACCAATGCAAAAAAATCAATAAAAAAATTAAATATAAAACAAGGAAAA  177
B07   GAAGCTTTTATTACCAATGCAAAAAAATCAATAAAAAAATTAAATATAAAACAAGGAAAA  177
B14   GAAGCTTTTATTACCAATGCAAAAAAATCAATAAAAAAATTAAATATAAAACAAGGAAAA  177
B24   GAAGCTTTTATTACCAATGCAAAAAAATCAATAAAAAAATTAAATATAAAACAAGGAAAA  177
B36   GAAGCTTTTATTACCAATGCAAAAAAATCAATAAAAAAATTAAATATAAAACAAGGAAAA  177
B46   GAAGCTTTTATTACCAATGCAAAAAAATCAATAAAAAAATTAAATATAAAACAAGGAAAA  177
W09   GAAGCTTTTATTACCAATGCAAAAAAATCAATAAAAAAATTAAATATAAAACAAGGAAAA  174
W20   GAAGCTTTTATTACCAATGCAAAAAAATCAATAAAAAAATTAAATATAAAACAAGGAAAA  174
W25   GAAGCTTTTATTACCAATGCAAAAAAATCAATAAAAAAATTAAATATAAAACAAGGAAAA  174
      ************************************************************
```

Figure S1
