# Supplementary material for: Differential Distribution of the wlaN and cgtB Genes, Associated with Guillain-Barré Syndrome, in Campylobacter jejuni Isolates from Humans, Broiler Chickens, and Wild Birds
Source: Microorganisms. 2020 Feb 26;8(3):325. doi: 10.3390/microorganisms8030325 (PMC7142995; doi:10.3390/microorganisms8030325)
Supplement: Supplementary file 1 [file FIG S3.pdf › FIG S3.pdf]

```
H58    AAAAGTTTGACTTTAATTCTGGTTTTTATAGCAAAAAAGAGTTTGTGAAAAAAAATTATTG  60
W17    AAAAGTTTGACTTTAATTCTGGTTTTTATAGCAAAAAAGAGTTTGTAAAAAAAAATTATTG  60
W32    AAAAGTTTGACTTTAATTCTGGTTTTTATAGCAAAAAAGAGTTTGTAAAAAAAAATTATTG  60
W34    AAAAGTTTGACTTTAATTCTGGTTTTTATAGCAAAAAAGAGTTTGTAAAAAAAAATTATTG  60
W36    AAAAGTTTGACTTTAATTCTGGTTTTTATAGCAAAAAAGAGTTTGTAAAAAAAAATTATTG  60
W37    AAAAGTTTGACTTTAATTCTGGTTTTTATAGCAAAAAAGAGTTTGTAAAAAAAAATTATTG  60
W39    AAAAGTTTGACTTTAATTCTGGTTTTTATAGCAAAAAAGAGTTTGTAAAAAAAAATTATTG  60
W40    AAAAGTTTGACTTTAATTCTGGTTTTTATAGCAAAAAAGAGTTTGTAAAAAAAAATTATTG  60
W43    AAAAGTTTGACTTTAATTCTGGTTTTTATAGCAAAAAAGAGTTTGTAAAAAAAAATTATTG  60
W44    AAAAGTTTGACTTTAATTCTGGTTTTTATAGCAAAAAAGAGTTTGTAAAAAAAAATTATTG  60
W46    AAAAGTTTGACTTTAATTCTGGTTTTTATAGCAAAAAAGAGTTTGTAAAAAAAAATTATTG  60
W47    AAAAGTTTGACTTTAATTCTGGTTTTTATAGCAAAAAAGAGTTTGTAAAAAAAAATTATTG  60
W49    AAAAGTTTGACTTTAATTCTGGTTTTTATAGCAAAAAAGAGTTTGTAAAAAAAAATTATTG  60
W02    AAAAGTTTGACTTTAATTCTGGTTTTTATAGCAAAAAAGAGTTTGTAAAAAAAAATTATTG  60
H37    AAAAGTTTGACTTTAATTCTGGTTTTTATAGCAAAAAAGAGTTTGTAAAAAAAAATTATTG  60
H60    AAAAGTTTGACTTTAATTCTGGTTTTTATAGCAAAAAAGAGTTTGTAAAAAAAAATTATTG  60
B04    AAAAGTTTGACTTTAATTCTGGTTTTTATAGCAAAAAAGAGTTTGTAAAAAAAAATTATTG  60
B06    AAAAGTTTGACTTTAATTCTGGTTTTTATAGCAAAAAAGAGTTTGTAAAAAAAAATTATTG  60
B41    AAAAGTTTGACTTTAATTCTGGTTTTTATAGCAAAAAAGAGTTTGTAAAAAAAAATTATTG  60
H61    AAAAGTTTGACTTTAATTCTGGTTTTTATAGCAAAAAAGAGTTTGTGAAAAAAAATTATTG  60
W10    AAAAGTTTGACTTTAATTCTGGTTTTTATAGTAAAAAAGAGTTTGTAAAAAAAAATTATTG  60
W15    AAAAGTTTGACTTTAATTCTGGTTTTTATAGTAAAAAAGAGTTTGTAAAAAAAAATTATTG  60
W18    AAAAGTTTGACTTTAATTCTGGTTTTTATAGCAAAAAAGAGTTTGTAAAAAAAAATTATTG  60
W27    AAAAGTTTGACTTTAATTCTGGTTTTTATAGCAAAAAAGAGTTTGTAAAAAAAAATTATTG  60
       ******************************* ************** *************

H58    CAAAGAAAAATTTATATTGGACTATGTGGGGGGAAACTTATAAGAAAGAAATTGTATTTA  120
W17    CAAAGAAAAATTTATATTGGACTATGTGGGG-GAAACTTATAAGAAAGAAATTGTATTTA  119
W32    CAAAGAAAAATTTATATTGGACTATGTGGGG-GAAACTTATAAGAAAGAAATTGTATTTA  119
W34    CAAAGAAAAATTTATATTGGACTATGTGGGG-GAAACTTATAAGAAAGAAATTGTATTTA  119
W36    CAAAGAAAAATTTATATTGGACTATGTGGGG-GAAACTTATAAGAAAGAAATTGTATTTA  119
W37    CAAAGAAAAATTTATATTGGACTATGTGGGG-GAAACTTATAAGAAAGAAATTGTATTTA  119
W39    CAAAGAAAAATTTATATTGGACTATGTGGGG-GAAACTTATAAGAAAGAAATTGTATTTA  119
W40    CAAAGAAAAATTTATATTGGACTATGTGGGG-GAAACTTATAAGAAAGAAATTGTATTTA  119
W43    CAAAGAAAAATTTATATTGGACTATGTGGGG-GAAACTTATAAGAAAGAAATTGTATTTA  119
W44    CAAAGAAAAATTTATATTGGACTATGTGGGG-GAAACTTATAAGAAAGAAATTGTATTTA  119
W46    CAAAGAAAAATTTATATTGGACTATGTGGGG-GAAACTTATAAGAAAGAAATTGTATTTA  119
W47    CAAAGAAAAATTTATATTGGACTATGTGGGG-GAAACTTATAAGAAAGAAATTGTATTTA  119
W49    CAAAGAAAAATTTATATTGGACTATGTGGGG-GAAACTTATAAGAAAGAAATTGTATTTA  119
W02    CAAAGAAAAATTTATATTGGACTATGTGGGG-GAAACTTATAAGAAAGAAATTGTATTTA  119
H37    CAAAGAAAAATTTATATTGGACTATGTGGGG-GAAACTTATAAGAAAGAAATTGTATTTA  119
H60    CAAAGAAAAATTTATATTGGACTATGTGGGG-GAAACTTATAAGAAAGAAATTGTATTTA  119
B04    CAAAGAAAAATTTATATTGGACTATGTGGGG-GAAACTTATAAGAAAGAAATTGTATTTA  119
B06    CAAAGAAAAATTTATATTGGACTATGTGGGG-GAAACTTATAAGAAAGAAATTGTATTTA  119
B41    CAAAGAAAAATTTATATTGGACTATGTGGGG-GAAACTTATAAGAAAGAAATTGTATTTA  119
H61    CAAAGAAAAATTTATATTGGACTATGTGGGG-GAAACTTATAAGAAAGAAATTGTATTTA  119
W10    CAAAGAAAAATTTATATTGGACTATGTGGGG-GAAGCTTATAAGAAAGAAATTGTATTTA  119
W15    CAAAGAAAAATTTATATTGGACTATGTGGGG-GAAGCTTATAAGAAAGAAATTGTATTTA  119
W18    CAAAGAAAAATTTATATTGGACTATGTGGGG-GAAGCTTATAAGAAAGAAATTGTATTTA  119
W27    CAAAGAAAAATTTATATTGGACTATGTGGGG-GAAGCTTATAAGAAAGAAATTGTATTTA  119
       ******************************* *** ************************

H58    GAAGCTTTTGCGAGTTTAAGACTCGAGAAAGATGTTAAAATCAATATGGCTGAAGATGTA  180
W17    GAAGCTTTTTCAAGTTTAAGACTCGAGAAAGATGTTAAAATCAATATGGCTGAAGATGTA  179
W32    GAAGCTTTTTCAAGTTTAAGACTCGAGAAAGATGTTAAAATCAATATGGCTGAAGATGTA  179
W34    GAAGCTTTTTCAAGTTTAAGACTCGAGAAAGATGTTAAAATCAATATGGCTGAAGATGTA  179
W36    GAAGCTTTTTCAAGTTTAAGACTCGAGAAAGATGTTAAAATCAATATGGCTGAAGATGTA  179
W37    GAAGCTTTTTCAAGTTTAAGACTCGAGAAAGATGTTAAAATCAATATGGCTGAAGATGTA  179
W39    GAAGCTTTTTCAAGTTTAAGACTCGAGAAAGATGTTAAAATCAATATGGCTGAAGATGTA  179
W40    GAAGCTTTTTCAAGTTTAAGACTCGAGAAAGATGTTAAAATCAATATGGCTGAAGATGTA  179
W43    GAAGCTTTTTCAAGTTTAAGACTCGAGAAAGATGTTAAAATCAATATGGCTGAAGATGTA  179
W44    GAAGCTTTTTCAAGTTTAAGACTCGAGAAAGATGTTAAAATCAATATGGCTGAAGATGTA  179
W46    GAAGCTTTTTCAAGTTTAAGACTCGAGAAAGATGTTAAAATCAATATGGCTGAAGATGTA  179
W47    GAAGCTTTTTCAAGTTTAAGACTCGAGAAAGATGTTAAAATCAATATGGCTGAAGATGTA  179
W49    GAAGCTTTTTCAAGTTTAAGACTCGAGAAAGATGTTAAAATCAATATGGCTGAAGATGTA  179
W02    GAAGCTTTTGCGAGTTTAAGACTCGAGAAAGATGTTACAATCAATATGGCTGAAGATGTA  179
H37    GAAGCTTTTGCGAGTTTAAGACTCGAGAAAGATGTTAAAATCAATATGGCTGAAGATGTA  179
H60    GAAGCTTTTGCGAGTTTAAGACTCGAGAAAGATGTTAAAATCAATATGGCTGAAGATGTA  179
B04    GAAGCTTTTGCGAGTTTAAGACTCGAGAAAGATGTTAAAATCAATATGGCTGAAGATGTA  179
B06    GAAGCTTTTGCGAGTTTAAGACTCGAGAAAGATGTTAAAATCAATATGGCTGAAGATGTA  179
B41    GAAGCTTTTGCGAGTTTAAGACTCGAGAAAGATGTTAAAATCAATATGGCTGAAGATGTA  179
H61    GAAGCTTTTGCGAGTTTAAGACTCGAGAAAGATGTTAAAATCAATATGGCTGAAGATGTA  179
W10    GAATCTTTTGCGAGTTTAAGACTCGAGAAAGATGTTAAAATCAATATGGCTGAAGATGTA  179
W15    GAATCTTTTGCGAGTTTAAGACTCGAGAAAGATGTTAAAATCAATATGGCTGAAGATGTA  179
W18    GAATCTTTTGCGAGTTTAAGACTCGAGAAAGATGTTAAAATCAATATGGCTGAAGATGTA  179
W27    GAATCTTTTGCGAGTTTAAGACTCGAGAAAGATGTTAAAATCAATATGGCTGAAGATGTA  179
       *** ***** * ************************ ***********************
```

Figure S3
